# The willingness and attitudes of speech-language pathologists towards the use of mobile health technology: a survey study

**DOI:** 10.1186/s12913-023-09339-1

**Published:** 2023-04-04

**Authors:** Soheila Saeedi, Marjan Ghazisaeedi, Maryam Ebrahimi, Mohammad-Sadegh Seifpanahi, Hamid Bouraghi

**Affiliations:** 1 Department of Health Information Technology, School of Allied Medical Sciences, Hamadan University of Medical Sciences, Hamadan, Iran; 2 Department of Health Information Management and Medical Informatics, School of Allied Medical Sciences, Tehran University of Medical Sciences, Tehran, Iran; 3 Department of Speech and Language Pathology, School of Rehabilitation Sciences, Hamadan University of Medical Sciences, Hamadan, Iran; 4 Department of Speech and Language Pathology, Autism Spectrum Disorders Research Center, School of Rehabilitation Sciences, Hamadan University of Medical Sciences, Hamadan, Iran

**Keywords:** Speech-language pathologists, Mobile health, mHealth, Willingness, Attitude

## Abstract

**Background:**

Mobile health (mHealth) technology could be used in different ways to treat various speech and language disorders. The attitude of speech-language pathologists (SLPs) towards this technology and their willingness to use it can play a significant role in the success of the therapies they provide. This study was conducted to investigate the willingness and attitude of SLPs towards the use of mHealth technology.

**Methods:**

This cross-sectional study was conducted from September 2021 to April 2022 in Iran. A researcher-made questionnaire consisting of three parts (information related to demographic variables, attitude and willingness) was designed based on the past studies, and then given to all SLPs throughout Iran. Data were analyzed by SPSS software, using descriptive and inferential statistics (frequency, mean, Fisher’s exact test, and analysis of variance). Also, the SLPs’ willingness to use the desired technology was interpreted as a percentage as follows: 0–20% = not at all willing, 21–40% = slightly willing, 41–60% = moderately willing, 61–80% = highly willing, and above 80% = extremely willing.

**Results:**

One hundred sixty speech-language pathologists from all over Iran participated in this study. The results showed that the willingness of 65.25% of SLPs to use the mentioned technology was at a good level, and according to the mentioned category, they had a high willingness to use this technology. In regard to the attitude of SLPs, the findings showed that SLPs believed that patients receive a higher quality of care during in-person visits than through mHealth technology. Also, this survey showed that SLPs were more inclined to use this technology to answer patients’ questions. Non-payment of services provided through mHealth technology and privacy concerns were the reasons for the lack of use of this technology by SLPs.

**Conclusions:**

SLPs are willing to use mHealth technology after solving the related challenges, including payment of costs and privacy concerns. However, SLPs believed that this technology will not be a suitable alternative to face-to-face sessions.

## Background

Health care systems highly depend on healthcare workers to provide care to patients. In many low- and middle-income countries, health care systems are severely understaffed or inequitably distributed to provide effective health care and promotion [1]. It is estimated that in 2030, the global demand for healthcare workers will reach 80 million, which is twice the number of current health workers, due to population growth and aging [2]. Due to the critical nature of service that the health system provides, the limited financial resources and the long time that the training of healthcare workers require, existing technologies can be used to help resolve these problems.

Mobile health technology can help solve the problems of staff shortages and the lack of patient access to health care services by establishing communication between health care providers and patients. Mobile health, also called mHealth, is a technology that uses telecommunication devices such as personal digital assistants, smartphones, laptops, and so on to provide health care and information.

In recent years, mHealth technology has been used for various purposes, which include (1) improving access to health services, (2) adherence to treatment, (3) management of chronic diseases, (4) education, monitoring, and communication with patient, and (5) reduction in burden of diseases caused by poverty [3–6]. A study conducted by Marcolino and colleagues on the impact of mHealth interventions shows the positive effect of this technology on improving the symptoms of asthmatic patients, helping to control blood sugar in diabetic patients, improving blood pressure, helping to lose weight in obese and overweight patients, improving disease symptoms, reducing hospitalization and mortality of patients with heart failure, and improving disease symptoms in patients with chronic lung. This study also showed that SMS reminders have the same effect as phone calls on increasing patient attendance, improving treatment adherence, and lowering the costs than other reminders [7].

Mobile health, an emerging mobile communication and network technology, has been able to provide health services to anyone, anywhere, and at any time by overcoming existing geographical, organizational and time barriers [8]. The adoption rate of mobile health technology in developed countries is different. This rate is estimated at 60% in developed countries with high income and 20% in middle and low-income countries. Also, the use of this technology is increasing in developing countries [9].

One of the areas in which mobile health technology can be used is language and speech disorders. This technology has had many applications in this field and has been used for different purposes. These tools have been used to diagnose speech-language disorders, as an auxiliary tool in the treatment process, and to send educational messages to the families of these patients [10–12]. Also, in a study that investigated the applications of mHealth technology to pediatric speech and language therapy, it was concluded that pediatric speech-language pathologists (SLPs) are using this technology for intervention (36.1%), clinical information (21.8%), parent education (13.7%), assessment (12%), client education (9.8%), and other uses (5.0%) [13]. Acceptance of this technology by SLPs can be constructive in the broad application of this technology in diagnosing and treating patients with speech and language disorders. It also paves the way for the rapid development of this technology in this field. Therefore, this study aimed to investigate the attitude of SLPs in Iran towards mHealth technology and their willingness to use it in the field of speech and language disorders.

## Methods

This analytical study was conducted between September 2021 and April 2022.

Speech therapy major in Iran includes three degrees: bachelor’s (BSc), master’s (MSc) and doctorate (PhD). The duration of a bachelor’s, master’s, and PhD degrees are 4, 2–3 and 4–5 years, respectively. High school graduates who can successfully pass the National University Entrance Examination can study for a bachelor’s degree in this field. The curriculum of the 4-year bachelor’s degree includes theoretical courses and clinical practice related to the all of the speech, language and swallowing disorder categories. The graduated BSc students, are certified by their universities and the Ministry of Health to do clinical practice. In Iran the BSc Speech therapy graduates can work as a clinician to evaluate and treat all patients with different speech, language, and swallowing disorders in private clinics, hospitals and university clinics, special needs schools and welfare organization. In the MSc and PhD, in addition to passing theoretical courses and clinical practice, students also carry out research projects related to their interested fields.

Speech-language pathologists from all over Iran participated in this study. The frequency distribution of respondents’ characteristics is displayed in Table 1. More than two-thirds of the participants in the study were female (66.9), and 104 of them (65%) had a bachelor’s degree. Among all SLPs, only one person (0.6%) did not have a smartphone, and other 99.4% had one. About 66% (n = 105) of the SLPs were working in private clinics and clinics supervised by universities of medical sciences. The age range of 65% of the participants in this study was between 20 and 29 years and all participants mean of 29.33 years (SD = 7.194). The minimum and maximum age of the participants were 20 and 56 years. One hundred and three participants (64.4%) had between 0 and 5 years of work experience, and their mean work experience was 6.29 years (SD = 6.4). The main age group of patients referred to SLPs were children (45%) and most patients had speech disorders (60%).

**Table 1:** Frequency distribution of speech-language pathologists’ characteristics

Variables	Groups	Number (%)
**Sex**	Female	107 (66.9)
	Male	53 (33.1)
**Education level**	Bachelor’s degree	104 (65)
	Masters’ degree	48 (30)
	Doctoral degree	8 (5)
**Having a cell phone**	Yes	159 (99.4)
	No	1 (0.6)
**type of cell phone**	No phone	1 (0.6)
	Simple phone	0 (0)
	Smart phone	159 (99.4)
**Workplace**	Private clinic	52 (32.5)
	Speech therapy clinics under the supervision of universities of medical sciences	53 (33.1)
	Speech therapy clinics of welfare organization	4 (2.5)
	Speech therapy clinics of special needs schools	4 (2.5)
	Speech therapy clinics of other organizations	16 (10)
	Work in more than one workplace	31 (19.4)
**Heard about “Mobile health”?**	Yes	113 (70.6)
	No	47 (29.4)
**Age**	20–29	104 (65)
	30–39	39 (24.4)
	40–49	14 (8.8)
	50–59	3 (1.9)
**Work experience**	0–5	103 (64.4)
	6–10	23 (14.4)
	11–15	18 (11.3)
	16–20	9 (5.6)
	21–25	5 (3.1)
	> 26	2 (1.3)
**Diagnostic categories of SLPs’ patients**	Speech disorders	96 (60)
	Language disorders	64 (40)
**Age range of SLPs’ patient caseloads**	Child (0–12)	72 (45)
	Adult (≥ 13)	24 (15)
	Mixed	64 (40)

In order to collect data, a researcher-made questionnaire (Tables 2 and 3) was designed by experts of medical informatics and cooperation of SLPs, based on relevant studies [9, 14–17]. The questionnaire consisted of three parts. The first part was related to demographic information, the second part was used to collect information related to the attitude of SLPs (19 questions), and the third part with 11 questions was related to the willingness of SLPs to use mHealth. A five-point Likert Scale, ranging from strongly agree to strongly disagree was used for the second and third parts of the questionnaire. In order to determine the face and content validity of the questionnaire, it was reviewed by five experts, and the results of their examination were used to revise the survey. The reliability of the questionnaire was tested using Cronbach’s alpha (0.726) in SPSS software.

**Table 2:** Mean score and frequency of SLPs’ attitude towards the use of mobile health technology

#	Questions	Strongly agree	Agree	Neutral	Disagree	Strongly disagree	Mean Score out of 4
		N (%)	N (%)	N (%)	N (%)	N (%)	
**1**	M-Health technology is easy to use.	60 (37.5)	71 (44.4)	21 (13.1)	7 (4.4)	1 (0.6)	3.14
**2**	Providing diagnostic, evaluation, and treatment services through mobile phones will be helpful for people with speech and language disorders.	48 (30)	84 (52.5)	24 (15)	4 (2.5)	0 (0)	3.1
**3**	The use of mobile health technology can be as effective as in-person visits and can be an alternative to them.	14 (8.8)	30 (18.8)	30 (18.8)	69 (43.1)	17 (10.6)	1.72
**4**	I will feel comfortable to monitor the health status of my patients through my mobile phone.	51 (31.9)	82 (51.2)	17 (10.6)	8 (5)	2 (1.3)	3.075
**5**	M-Health technology makes it possible to remind patients of medical orders (practices or education).	61 (38.1)	84 (52.5)	10 (6.3)	4 (2.5)	1 (0.6)	3.25
**6**	M-Health technology gives me the opportunity to, if necessary, modify the therapeutic exercises for my patient.	54 (33.8)	81 (50.6)	17 (10.6)	8 (5)	0 (0)	3.13
**7**	The use of mHealth technology can increase patients’ adherence to therapeutic exercises at home.	39 (24.4)	77 (48.1)	23 (14.4)	20 (12.5)	1 (0.6)	2.83
**8**	M-Health technology can establish an effective relationship between patients and SLPs to monitor their health status.	41 (25.6)	84 (52.5)	25 (15.6)	9 (5.6)	1 (0.6)	2.97
**9**	Using this technology will increase my efficiency and effectiveness.	40 (25.0)	78 (48.8)	26 (16.3)	14 (8.8)	2 (1.3)	2.875
**10**	Using this technology will improve the process of patient treatment.	34 (21.3)	79 (49.4)	34 (21.3)	11 (6.9)	2 (1.3)	2.825
**11**	M-Health technology will improve the delivery of care in remote and rural areas.	68 (42.5)	67 (41.9)	16 (10.0)	8 (5.0)	1 (0.6)	3.21
**12**	Using this technology will lead to the establishment of justice in patients’ access to health services.	50 (31.3)	60 (37.5)	36 (25.5)	13 (8.1)	1 (0.6)	2.91
**13**	Confidentiality of patient information is maintained during the use of mobile devices.	68 (42.5)	51 (31.9)	30 (18.8)	11 (6.9)	0 (0)	3.1
**14**	M-health can help reduce the costs of patient treatment.	46 (28.7)	65 (40.6)	34 (21.3)	14 (8.8)	1 (0.6)	2.88
**15**	I think that if I use this technology, most patients will also use this technology.	30 (18.8)	53 (33.1)	48 (30.0)	28 (17.5)	1 (0.6)	2.52
**16**	Privacy concerns are not a barrier to the use of this technology.	8 (5.0)	42 (26.3)	50 (31.3)	48 (30.0)	12 (7.5)	1.9
**17**	M-health technology can help me to repeat the necessary training and exercises frequently and if needed by the patients.	50 (31.3)	88 (55.0)	17 (10.6)	5 (3.1)	0 (0)	3.14
**18**	The use of mHealth technology helps me to devote more time to the patients than visiting them in-person.	24 (15.0)	52 (32.5)	29 (18.1)	43 (26.9)	12 (7.5)	2.21
**19**	The quality of services using mHealth technologies will not be much different compared to in-person visits.	11 (6.9)	31 (19.4)	30 (18.8)	69 (43.1)	19 (11.9)	1.66
**Total**							2.76

**Table 3:** Mean score and frequency of willingness to use mobile health technology by SLPs

#	Questions	Strongly agree	Agree	Neutral	Disagree	Strongly disagree	Mean Score out of 4
		N (%)	N (%)	N (%)	N (%)	N (%)	
**1**	If I can answer patients’ questions through mobile applications, I will be interested in using it “in addition to in-person visiting.“	78 (48.8)	65 (40.6)	7 (4.4)	9 (5.6)	1 (0.6)	3.31
**2**	If I can answer patients’ questions via cell phone, I would be interested in using a cell phone “instead of an in-person appointment.“	31 (19.4)	44 (27.5)	22 (13.8)	55 (34.4)	8 (5)	2.22
**3**	I would like to use the mobile phone more in “diagnosing” disorders.	29 (18.1)	59 (36.9)	34 (21.3)	29 (18.1)	9 (5.6)	2.44
**4**	I would like to use the mobile phone more in “treating” disorders.	29 (18.1)	73 (45.6)	36 (22.5)	18 (11.3)	4 (2.5)	2.66
**5**	I will use m-health technology if it is free for speech therapists.	54 (33.8)	70 (43.8)	18 (11.3)	14 (8.8)	4 (2.5)	2.975
**6**	Failure to pay for treatment provided via mobile phone does not reduce the use of this technology.	6 (3.8)	29 (18.1)	37 (23.1)	71 (44.4)	17 (10.6)	1.6
**7**	lack of knowledge about the use of this technology is not an obstacle to its use.	14 (8.8)	59 (36.9)	27 (16.9)	46 (28.7)	14 (8.8)	2.08
**8**	If patients accept this technology, I will be more willing to use these technologies.	48 (30.0)	92 (57.5)	11 (6.9)	7 (4.4)	2 (1.3)	3.11
**9**	I am willing to use this technology even if there is no obligation from the Ministry of Health.	16 (10.0)	79 (49.4)	35 (21.9)	23 (14.4)	7 (4.4)	2.46
**10**	If the Ministry of Health passes the law to reimburse the costs of providing services through mHealth technologies, I will be willing to use it.	26 (16.3)	84 (52.5)	36 (22.5)	12 (7.5)	2 (1.3)	2.75
**11**	If mHealth technologies are implemented, I will use them.	49 (30.6)	89 (55.6)	13 (8.1)	7 (4.4)	2 (1.3)	3.1
**Total**							2.61

In order to collect information, we provided the questionnaire to the SLPs participating in the study. The questionnaire was designed electronically in Porsline, and its link was sent via email to SLPs. Also, the questionnaire’s link was provided to the SLPs through social networks such as Telegram and WhatsApp, and they were asked to complete the questionnaire if they wished to do so. During the 8 months that the data collecting was carried out, the SLPs were given the option of completing the questionnaire.

In order to analyze the data and calculate the mean score for each question, the 5-point Likert scale was set as follow: Strongly agree = 4, agree = 3, neutral = 2, disagree = 1, and strongly disagree = 0. Based on this scoring, the mean score was calculated for each question. Descriptive statistics (frequency and mean) and inferential statistics (Fisher’s exact test and analysis of variance) were performed through IBM SPSS software. Also, the willingness to use mHealth was interpreted as following percentages: 0–20% indicating not at all willing, 21–40% indicating slightly willing, 41–60% indicating moderately willing, 61–80% showing highly willing, and above 80% indicating extremely willing [14].

## Results

A total of 160 questionnaires were completed by speech-language pathologists. There were no missing data in the completed questionnaires; in the design of the electronic questionnaire, it was necessary to answer all questions.

### SLP’s attitude towards the use of mHealth technology

The mean score and frequency distribution of attitude questions are shown in Table 2. The total mean score of SLPs’ attitudes was 2.76 out of 4. The lowest score was related to question 19, which was related to the quality of services provided through mHealth technology and in-person visits, with a score of 1.66 out of 4. The highest score was related to question 5, which was related to reminders of medical orders to patients by this technology, with a score of 3.25. The other answers that received low scores were related to the attitude of SLPs towards the use of mHealth technology including: (1) “Can mobile health technology be as effective as in-person visits and can it be an alternative to such visits?“ and (2) “Are privacy concerns a barrier to the use of this technology?“

### SLP’s willingness to use mHealth technology

Table 3 shows the frequency and mean score of participants’ willingness to use mHealth. The total mean score of SLPs’ willingness was 2.61 out of 4 (65.25%), which indicates that the participants had a high willingness to use mHealth technology. The lowest score of willingness (1.6) was related to “the impact of not paying for services provided through smartphones on the level of SLPs’ willingness to use this technology.“ In fact, the SLPs indicated that not paying for the services could reduce their willingness to use mHealth technology. Most SLPs (89.4%) were willing to use mHealth technology in addition to in-person visits to answer patients’ questions. Also, 87.5% of SLPs indicated that the acceptance of mHealth technology by patients could affect their willingness to use it. Furthermore, 82.5% of the participants confirmed the helpfulness of providing diagnostic, evaluation, and treatment services through mobile phones for people suffering from speech and language disorders.

Table 4 shows the relationship between demographic variables and “SLPs’ willingness to use mHealth technology”. According to Fisher’s exact test, there was no significant relationship between gender and the participants’ responses to questions (P = 0.245). Generally, it can be stated that the responses of men and women who answered the question related to the “use of mHealth technologies” were the same. Additionally, this test showed no significant difference between education level and “use of mHealth technologies” (P = 0.321). In all three groups of respondents with bachelor’s degrees (55.8%), master’s degrees (50%), and doctorates (55.6%), the highest response rate to the question about the “use of mHealth technologies” was related to the “agree” option.

**Table 4:** The relationship between demographic variables and “willingness to use mHealth technology” according to the Fisher’s exact test

Variables	Variable’s level	Use of mHealth technology if implemented					P-value
		Strongly agree	Agree	Neutral	Disagree	Strongly disagree	
**Sex**	Female	32 (29.9)	59 (55.1)	11 (10.3)	5 (4.7)	0 (0.0)	0.245
	Male	17 (32.1)	30 (56.6)	2 (3.8)	2 (3.8)	2 (3.8)	
**Education level**	Bachelor’s degree	30 (28.8)	58 (55.8)	10 (9.6)	6 (5.8)	0 (0.0)	0.321
	Masters’ degree	18 (37.5)	24 (50)	3 (6.2)	1 (2.1)	2 (4.2)	
	Doctoral degree	1 (12.5)	7 (87.5)	0 (0.0)	0 (0.0)	0 (0.0)	
**Owning a cell phone**	Yes	48 (30.2)	89 (56)	13 (8.2)	7 (4.4)	2 (1.2)	0.444
	No	1 (100)	0 (0.0)	0 (0.0)	0 (0.0)	0 (0.0)	
**Type of cell phone**	No phone	1 (100)	0 (0.0)	0 (0.0)	0 (0.0)	0 (0.0)	0.444
	Simple phone	0 (0.0)	0 (0.0)	0 (0.0)	0 (0.0)	0 (0.0)	
	Smart phone	48 (30.2)	89 (56)	13 (8.2)	7 (4.4)	2 (1.2)	
**Heard about “Mobile health”?**	Yes	40 (35.4)	60 (53.1)	8 (7.1)	3 (2.7)	2 (1.8)	0.113
	No	9 (19.1)	29 (61.7)	5 (10.6)	4 (8.5)	0 (0.0)	
**Workplace**	Private clinic	16 (30.8)	30 (57.7)	3 (5.8)	3 (5.8)	0 (0.0)	0.255
	Speech therapy clinics under the supervision of universities of medical sciences	19 (35.8)	31 (58.5)	3 (5.7)	0 (0.0)	0 (0.0)	
	Speech therapy clinics of welfare organization, special needs schools and other organizations	5 (20.8)	14 (58.3)	2 (8.3)	2 (8.3)	1 (4.2)	
	Work in more than one workplace	9 (29)	14 (45.2)	5 (16.1)	2 (6.5)	1 (3.2)	
**Age range of SLPs’ patient caseloads**	Child (0–12)	35 (48.6)	27 (37.5)	6 (8.3)	4 (5.6)	0 (0.0)	0.001
	Adult (≥ 13)	2 (8.3)	14 (58.3)	3 (12.5)	3 (12.5)	2 (8.3)	
	Mixed	12 (18.8)	48 (75.0)	4 (6.3)	0 (0.0)	0 (0.0)	
**Diagnostic categories of SLPs’ patients**	Speech disorders	31 (32.3)	53 (55.2)	6 (6.3)	6 (6.3)	0 (0.0)	0.209
	Language disorders	18 (28.1)	36 (56.3)	7 (10.9)	1 (1.6)	2 (3.1)	

According to Fisher’s exact test, there was no significant difference between having or not having a smartphone and the type of smartphone with “the use of mHealth technologies” (P = 0.444). No significant relationship was also observed between “heard about mHealth?“ and the “use of mHealth technologies” (P = 0.113). There was no significant difference between the workplace and the “use of mHealth technologies” (P = 0.255). The agreement rate for the “use of mHealth technologies” was 88.5% for the private clinic, 94.3% for speech therapy clinics under university supervision, 79.1% for speech therapy clinics of welfare organizations, special needs schools, and other organizations, and 74.2% for SLPs who work in more than one organization.

Finally, according to Fisher’s exact test, there was significant difference between the “age range of SLPs’ patient caseloads” with the “use of mHealth technologies” (P < 0.001). However, no significant relationship was observed between “diagnostic categories of SLPs’ patients” and the “use of mHealth technologies” (P = 0.209).

The analysis of variance test was used to compare the mean scores between the variables of age, work experience and the “use of mobile health technologies”. According to the results of this test, there was no significant relationship between SLPs’ age and “the use of m-health technologies” (P = 0.993), and between years of work experience and “use of m-health technologies” (P = 0.993).

## Discussion

This study was conducted to determine the willingness and attitudes of speech-language pathologists towards the use of mHealth technology. One hundred sixty researcher-made questionnaires were completed and analyzed. The results showed that 65.25% of SLPs were willing to use the mentioned technology. After examining the attitude of SLPs towards the use of mHealth technology, we found that the quality of services provided through in-person visits and mHealth technology is not the same according the respondents. They also indicated that privacy concerns could be a cause to prevent SLPs from using this technology. Also, this survey showed that SLPs were more inclined to use this technology to answer patients’ questions. They also declared that the acceptance of this technology by patients could lead to an increase in their willingness to use it. Regards to the purpose of SLPs using mHealth technology, most of the SLPs asserted its applicability to diagnose, assess, and treat people with speech and language disorders.

The results of this study revealed that the SLPs’ willingness to use mHealth technology was about 65%. In a study, Alazzam and colleagues [17] investigated the willingness of Gynecologists to use mobile health applications. The results of their study indicated that 79.12% of responders were willing to use this technology and similar to our study, their willingness was at a good level. A related study [14] also showed that the willingness of 70% of gynecologists to use this technology was at a good level. Among the reasons why the willingness to use this technology among SLPs was not at an excellent level, we can argue that around 30% of the SLPs responding to the questionnaire were not adequately familiar with this technology and had not heard about it. Around 18% of these SLPs also reported that using this technology was not easy for them. Not knowing how to charge for the services provided through this technology in Iran was also another factor that contributed to this problem. As the SLPs indicated in the survey, the potential free-of-charge nature of services provided by this technology was a serious obstacle to their willingness to use this technology. It seems that to increase the healthcare professionals’ willingness to use mHealth, it is necessary to properly introduce its applications and benefits to SLPs.

Among the factors that led to the reduction of SLPs’ willingness to use mobile health technology, the most important was the concern about the free of charge nature of services provided by this technology or paying less than in-person visits. In Iran, the patient pays all the costs of speech therapy services, and these costs are not reimbursed by insurance companies. Considering that the provision of speech therapy services by mobile health technology is not common in this country, most patients are not familiar with this technology and are not sure of its effectiveness. As a result, many patients resist paying these fees before ensuring its effectiveness or are willing to pay a much lower fee than in-person sessions. Also, the payment methods and channels for the services provided by this technology are not clear in Iran. Therefore, according to the reasons mentioned above, several issues can cause concern among health care providers about the cost of services, which include an unclear method of payment for the services provided in this way, a lack of specific policy guidelines to pay for the services in this way, and problems related to the non-inclusion of these services in insurance coverage. Therefore, health organizations and insurers should provide clear guidelines for payment and reimbursement costs, so that healthcare providers can provide health services through mHealth technology with more certainty.

On the other hand, the willingness of patients to pay for these services should also be considered. A study in this field concluded that patients are willing to pay if the mHealth technology is beneficial for them and improves their health [18].

This study showed that 53.75% of SLPs indicated that the effectiveness of services provided through mHealth technology is not equal to in-person consultations and cannot be a substitute for to in-person visits. SLPs indicated they tend to restrict the use of mHealth technology to answering patients’ questions. In recent years, mHealth technology has become a communication tool between physicians and patients and has led to the exchange of information between them [19]. Digital communication can improve patients’ satisfaction as, in some instances, it reduces the need for face-to-face visits, for example when a patient needs to ask a question of a non-medical nature [19–22].

Another concern of SLPs regarding the use of mHealth was protecting patients’ privacy while using this technology. Due to the sensitivity of health data, this point has always been raised in the health field. In the review studies that have been carried out in this regard, different suggestions have been presented in order to protect privacy, including analysis of the destination, analysis of the content of communications, use of remote storage, protection of systems and information, authentication, access control, individual participation, and privacy authorization [23, 24].

## Conclusions

This study showed that the willingness to use mHealth technology among SLPs was high. These specialists were more inclined to use mHealth technology as an addition to, but not as a replacement for, face-to-face consultations. The use of mHealth technology in this field faces many challenges, including safeguarding patients’ privacy and the need to create clear policy guidelines for payment processes that should be resolved by mHealth technology developers and the Ministry of Health. It is recommended that the developers of mHealth technology involve SLPs in all stages of development and use their views in the analysis stage and the development of mHealth applications should be based on a safe platform. Also, it is recommended that future studies investigate organization-level factors that can influence the willingness of SLPs.

## Data Availability

All data generated or analyzed during this study are included within this article.

## List of abbreviations

mHealth: Mobile health
SLPs: speech-language pathologists
